# Olfactory Network Disruptions as Mediators of Cognitive Impairment in De Novo Parkinson's Disease

**DOI:** 10.1111/cns.70198

**Published:** 2025-01-13

**Authors:** Yi Xing, Hao Zhou, Shuoying Chen, Yajie Wang, Jingru Ren, Yiting Cao, Jingzhe Li, Weiguo Liu

**Affiliations:** ^1^ Department of Neurology The Affiliated Brain Hospital of Nanjing Medical University Nanjing China; ^2^ Department of Neurology The First People's Hospital of Yancheng Yancheng China

**Keywords:** cognitive impairment, NBS, olfactory dysfunction, olfactory network, Parkinson's disease

## Abstract

**Objectives:**

Parkinson's disease (PD) is characterized by olfactory dysfunction (OD) and cognitive deficits at its early stages, yet the link between OD and cognitive deficits is also not well‐understood. This study aims to examine the changes in the olfactory network associated with OD and their relationship with cognitive function in de novo PD patients.

**Methods:**

A total of 116 drug‐naïve PD patients and 51 healthy controls (HCs) were recruited for this study. Graph theoretical approaches were employed to reveal the abnormalities of topological characteristics in the olfactory network. Network‐based statistics (NBS) analysis was employed to identify the abnormal subnetworks within the olfactory network. Moreover, partial correlation analysis and mediation analysis were performed to examine the relationship between the abnormal network metrics, olfactory function, and cognitive function.

**Results:**

Graph theoretical approaches revealed reduced betweenness centrality of the left insula in PD patients with OD. NBS analysis identified a disrupted subnetwork with decreased functional connectivity, primarily involving limbic regions. The average functional connectivity of this subnetwork partially mediated the relationship between olfactory and cognitive performance. Higher‐granularity network analysis further highlighted the insula's key role and revealed reduced efficiency of information integration within the olfactory network.

**Conclusions:**

OD was associated with specific changes in the functional olfactory network, which, in turn, affects cognitive function. These findings underscore the importance of assessing and addressing OD. Understanding the neural correlates of OD could provide novel insights into the management and comprehension of cognitive impairment in PD.

## Graphical abstract

A disrupted subnetwork of human olfactory network was observed in PD patients with olfactory dysfunction, and the average functional connectivity of this subnetwork partially mediates the effects of olfactory function on global cognitive function.

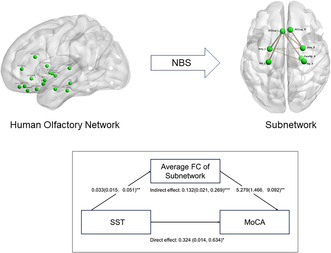

## 1 Introduction

Parkinson's disease (PD) is a prevalent neurodegenerative disorder, second only to Alzheimer's disease (AD). Olfactory dysfunction (OD) and cognitive impairment are two critical non‐motor symptoms in the early stages of PD [1]. Indeed, OD has consistently been regarded as a bellwether for the development of cognitive impairment and neurodegenerative diseases [2]. In diseases predominantly characterized by cognitive impairment, such as AD, the relationship between olfactory and cognitive functions has been well established [3, 4]. A similar relationship has also been observed in PD [5, 6, 7]. Moreover, previous research indicated that PD patients with cognitive decline were likely to experience more severe OD as the disease progresses [8]. However, the exact neuropathological mechanisms underlying this reciprocal relationship remain poorly understood.

According to the olfactory vector hypothesis [9], PD‐related α‐synuclein pathology may propagate through the olfactory system, with various brain regions along this pathway playing essential roles in both olfactory and cognitive functions. Previous neuroimaging studies have provided valuable insights into the neural mechanisms linking OD and cognitive impairment in neurodegenerative diseases. In AD and mild cognitive impairment (MCI), structural magnetic resonance imaging (MRI) studies demonstrated gray matter atrophy in olfactory‐related regions, including the hippocampus, amygdala, parahippocampal gyrus, and entorhinal cortex [10], which are also critical for cognitive processes. Similar patterns of gray matter atrophy were also observed in PD, with longitudinal atrophy of the hippocampus and amygdala being associated with cognitive impairment [11, 12, 13]. Furthermore, PD patients with MCI exhibited poorer olfactory functioning and reduced white matter integrity in the anterior olfactory structures [14]. In functional neuroimaging studies, individuals with hyposmia showed less activation in the right orbitofrontal cortex, amygdala, and left anterior cingulate cortex (ACC) compared to those without hyposmia [15]. Additionally, a study utilizing data‐driven independent component analysis identified several olfaction‐related brain regions, including the piriform cortex, insula, thalamus, and orbitofrontal cortex, with notable decreases in activation observed in the bilateral insula and right orbitofrontal cortex in PD patients [16]. Furthermore, olfactory impairment in PD was associated with decreased functional connectivity (FC) of the hippocampus and amygdala, which correlated with both cognitive and olfactory performances [11, 17]. These findings from structural and functional MRI studies highlighted the intricate relationship between OD and cognitive impairment in PD. However, most of these studies have focused primarily on one or a few brain regions, and few have investigated this relationship from a brain network perspective, particularly in patients with PD.

In fact, the human olfactory network can be divided into three subnetworks, with only one subnetwork involved in pure olfactory perception, while the other two extensively contribute to higher‐level neural functions such as cognition and emotion [18]. Carnemolla et al. found that in patients with AD and frontotemporal dementia [19], the volumes of olfactory network regions significantly reduced. Moreover, OD associated with both COVID‐19 [20] and diabetes [21, 22] was linked to disruptions in the olfactory network, which, in turn were correlated with declines in cognitive performance. While these studies have preliminarily explored the role of the olfactory network in olfactory and cognitive functions, the specific alterations of the network in early‐stage PD patients with OD remain unclear and require further investigation.

Given the strong correlation between olfactory and cognitive functions, as well as the existing research gap regarding the influence of olfactory networks on cognition in patients with PD, the aim of this study was to examine the alterations in the functional olfactory network in PD patients with OD. Additionally, the effects of these abnormal network changes on cognitive function were examined. We hypothesize that alterations in the olfactory network triggered by OD in early‐stage PD patients are also linked to cognitive function.

## 2 Materials and Methods

### 2.1 Participants

A total of 116 drug‐naive PD patients were recruited from the Affiliated Brain Hospital of Nanjing Medical University, along with 51 healthy controls (HCs) matched for age, sex, and education. Three PD patients were excluded from the study due to excessive head motion (cumulative translation or rotation > 3.0 mm or 3.0°). A total of 113 patients diagnosed with PD and 51 neurologically HCs were finally included in the data analysis. The inclusion criteria for PD patients were as follows: (1) aged between 40 and 80 years; (2) PD diagnosis based on the United Kingdom Parkinson's Disease Society Brain Bank Criteria; (3) no history of prior treatment with anti‐PD medications, and (4) a minimum of 1‐year clinical follow‐up. The HCs were neurologically normal. Exclusion criteria for all participants included: (1) history of cerebral function‐affecting conditions (e.g., brain damage, cerebrovascular disease, white matter disease, prior brain surgery); (2) serious illnesses (e.g., cancer, diabetes, hyperthyroidism, severe psychiatric and systemic disorders); (3) dementia or inability to complete neuropsychological tests or MRI scans; and (4) use of specific medications (e.g., anticholinergic drugs, antidepressants). Demographic information, including sex, age, age of onset, and years of education, was systematically collected from all enrolled patients as well as participants in the control group.

The Medical Ethics Committee of the Affiliated Brain Hospital of Nanjing Medical University provided ethical approval for this study. Written informed consent was obtained from all the patients before study participation.

### 2.2 Olfactory Functional Assessment

The study employed the Sniffin’ Sticks test (SST), which comprises 12 odor pens representing 12 test items, each accompanied by 4 multiple‐choice options. Participants identified the presented odor by selecting the correct option after smelling the pen placed approximately 3–5 cm in front of their noses. Correct answers were awarded one point, with a maximum possible score of 12 points. Patients scoring greater than or equal to 8 were assigned to the PD without OD group (NOD, *n* = 36), while the remaining patients were assigned to the PD with OD group (OD, *n* = 77) [23, 24]. Among the HC group, 40 participants were evaluated using the SST, and all exhibited normal olfactory function. The remaining 11 HCs reported no subjective hyposmia during our screening protocol for common symptoms associated with prodromal PD and were also included in the subsequent analysis.

### 2.3 Clinical Evaluation

The motor symptoms of PD patients were assessed using the Unified Parkinson's Disease Rating Scale Part III (UPDRS‐III) and Hoehn and Yahr (H − Y) stage. The non‐motor symptoms were evaluated using the Parkinson's Disease Non‐Motor Symptoms Questionnaire (PDNMS). The Hamilton Depression Rating Scale (HAMD) and the Hamilton Anxiety Scale (HAMA) were employed to quantify depression and anxiety.

As recommended by the Movement Disorder Society (MDS), [25] the Montreal Cognitive Assessment (MoCA) was employed to assess the global cognitive function of all participants. To further analyze the correlation between neuroimaging parameters and specific cognitive domains, 82 of the 113 PD patients in this study underwent a formal and comprehensive neuropsychological evaluation that encompassed five cognitive domains: attention/working memory [Digit Span Backward Test (DST) and Stroop Color‐Word Test (SCWT)], executive [Trail Making Test B(TMT‐B), verbal fluency test (VFT) and Clock Drawing Test (CDT)], visuospatial [Benton's Judgment of Line Orientation Test (JLOT) and Hooper Visual Organization Test (HVOT)], memory [Auditory Verbal Learning Test (AVLT) and Logical Memory Test (LMT)], and language [Boston Naming Test (BNT) and Wechsler Adult Intelligence Scale III(WAIS‐III) Similarities Test]. Moreover, the scores of each test were transformed into z‐scores and their orientations were standardized, ensuring that higher scores reflect better performance in the respective cognitive domain. Subsequently, the average score of all tests within each cognitive domain was computed, representing the comprehensive score for that specific cognitive domain [26].

### 2.4 MRI Data Acquisition and Preprocessing

Axial anatomical images were acquired using a 3D‐T1 fluid attenuated inversion recovery sequence. The scan parameters were set as follows: repetition time = 2530 ms; echo time = 3.34 ms; flip angle = 7°; matrix = 256 × 192; field of view = 256 × 256 mm; slice thickness/gap = 1.33/0.5 mm; bandwidth = 180 HZ/PX; 128 slices covered the whole brain. Resting‐state functional magnetic resonance imaging (rs‐fMRI) were subsequently collected in the same slice orientation with a gradient‐recalled echo‐planar imaging pulse sequence, which included 240 volumes. The parameters were: repetition time = 2000 ms; echo time = 30 ms; flip angle = 90°; matrix = 64 × 64, field of view = 220 × 220 mm; thickness/gap = 3.5/0.6 mm; bandwidth = 2232HZ/PX.

All rs‐fMRI data were preprocessed using the Resting‐State fMRI Data Analysis Toolkit plus V1.27 (RESTplus V1.27, http://restfmri.net/forum/RESTplus), which is based on Statistical Parametric Mapping (SPM12, https://www.fil.ion.ucl.ac.uk) and implemented in MATLAB R2018b (http://www.mathworks.com/products/matlab). The preprocessing pipeline included the following steps: (1) removal of the first 10 time points; (2) slice timing correction; (3) head motion correction, and participants with excessive head motion (cumulative translation or rotation > 3.0 mm or 3.0°) were excluded; (4) normalization of the EPI images to the Montreal Neurological Institute (MNI) standard space using T1 image unified new segmentation, and resampling to an isotropic voxel size of 3 mm; (5) in nuisance covariate regression, linear trends, 24 parameters of Friston's head motion model, white matter signal, and cerebrospinal fluid signal were selected as nuisance covariates; and (6) residuals were bandpass filtered (0.01–0.08 Hz).

### 2.5 Functional Brain Network Analyses

**FIGURE 1:**
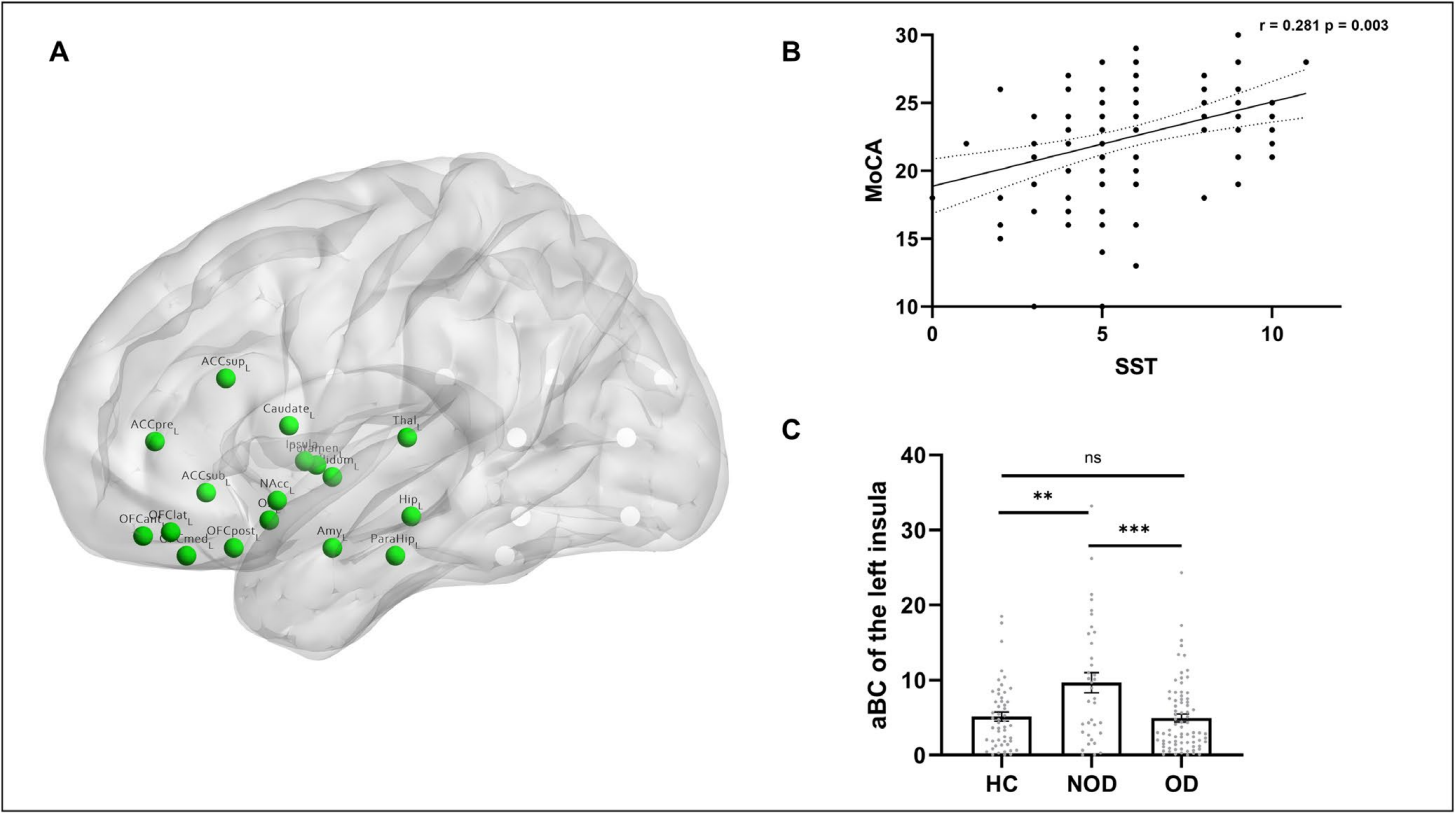
(A) Unilateral 17 nodes in olfactory network: Olfactory cortex, Anterior cingulate cortex pregenual (ACCpre), Anterior cingulate cortex subgenual (ACCsub), Anterior cingulate cortex supracollasal (ACCsup), Amygdala, Caudate nucleus, Hippocampus, Nucleus Accumbens (NAcc), nucleus Pallidum, ParaHippocampal gyrus, Putamen, Anterior orbital gyrus (OFCant), Lateral orbital gyrus (OFClat), Medial orbital gyrus (OFCmed), Posterior orbital gyrus (OFCpost), Insula and Thalamus. (B) The correlation between the MoCA and SST scores among PD patients. (C) The intergroup differences in aBc of the left insula. *p < 0.05, **p < 0.01, **p < 0.001.

Based on previous neuroimaging studies of human olfactory‐related brain regions [20, 21, 27, 28], we selected 34 brain regions associated with olfaction from the Automated Anatomical Labeling version 3 (AAL3) atlas [29] to serve as nodes of the olfactory network (Figure 1A, Analysis Part I). To further investigate the subtle changes within the olfactory network of PD patients with OD, the Brainnetome Atlas [30] was used to subdivide the aforementioned olfactory‐related brain regions to construct an olfactory network with higher granularity (Table S1 and Figure S6A, Analysis Part II). Subsequently, the FC values between these brain regions were calculated using the GRETNA toolbox, and these values were defined as the edges of the network. This process resulted in a FC matrix of either 34 × 34 or 78 × 78 dimensions. Finally, Fisher's z‐transformation was performed to convert the data into *z*‐values that approximate a normal distribution.

To validate whether the aforementioned olfactory‐related brain regions are connected to one another, an average FC matrix was computed for all participants and for each group separately. A one‐sample *t*‐test was subsequently performed for each connection to ascertain whether it significantly deviated from zero, with Bonferroni correction applied to control for multiple comparisons. Both significantly positive and significantly negative connections were identified and quantified for each analyzed group.

Sparsity is defined as the ratio of the actual number of edges in the network to the maximum possible number of edges. The lower limit of sparsity is critical for ensuring network connectivity and facilitating an effective analysis of topological structure. This limit was estimated using the built‐in functions of the GRETNA toolbox, with values set at 0.10 and 0.06 for Part I and Part II Analyses, respectively. Subsequently, the small‐worldness (σ) for each participant was calculated across a range of sparsity values, from the lower limit up to 0.50, with increments of 0.01. To confirm the presence of small‐world properties within the olfactory networks, the upper limit of sparsity was defined as the threshold at which σ ≥ 1.1 for more than 95% of the participants' individual olfactory networks. Therefore, the topological properties were calculated at each sparsity level (0.10–0.38, 0.06–0.30, for Part I and Part II Analyses, respectively, at steps of 0.01): global metrics of normalized clustering coefficient (γ), normalized characteristic path length (λ), σ, characteristic path length (Lp), characteristic clustering coefficient (Cp), global efficiency (Eglob) and local efficiency (Eloc), and the nodal metrics included betweenness centrality (Bc), degree centrality (Dc), clustering coefficient (NCp), nodal efficiency (Ne), and nodal local efficiency (NLe).

Finally, to further identify functional subnetworks manifesting differences between each pair of groups, we utilized network‐based statistics (NBS) analysis, which is a nonparametric statistical approach for controlling family‐wise errors in connection analyses.

### 2.6 Statistical Analyses

Statistical analyses were conducted using SPSS version 25 (SPSS Inc.). The Kolmogorov–Smirnov test was utilized to evaluate the normality of the data. Continuous variables were reported as means ± standard deviations (SDs), while categorical variables were presented as counts (%). Demographic and clinical variables were compared using the Mann–Whitney U test, two‐sample *t*‐test, Kruskal–Wallis W test, analysis of variance (ANOVA), or chi‐squared test. The Bonferroni correction was applied for post hoc comparisons.

Analysis of covariance (ANCOVA) was used to compare the area under the curve (AUC) of network topological properties among the three groups based on the GRETNA toolbox, with age, sex, and years of education as covariates. For the node metrics, the false discovery rate (FDR) correction method was applied to reduce the risk of false‐positive errors due to multiple comparisons. Furthermore, the Bonferroni correction was applied for post hoc pairwise comparisons of the topological properties identified by ANCOVA.

In the NBS analysis, we first conducted a two‐sample *t*‐test on each edge independently to test for significant differences in connectivity values between two groups. A primary edge threshold (*p* = 0.01) was then applied to identify a set of edges exceeding this threshold, among which connected components and their sizes could be determined. The statistical significance of the observed component sizes was then evaluated by comparing them to an empirical null distribution of maximal component sizes, obtained under the null hypothesis of random group membership (5000 permutations). Subnetworks that showed significance at a corrected level of *p* = 0.05 were reported, after adjusting for the effects of age, sex, and years of education [31].

Subsequently, within the PD group, we examined the partial correlation between clinical measurements (UPDRS‐III, SST, MoCA, and the mean *z*‐scores of each cognitive domain) and neuroimaging parameters (nodal metrics and average FC of the identified subnetworks in NBS analysis) that showed significant inter‐group differences, adjusting for the effects of age, sex, years of education, and disease duration. Considering that certain nodes of the olfactory network belong to the limbic system, HAMA and HAMD were also included as additional covariates. Statistical significance was set at *p*‐value < 0.05. Mediation analysis was performed using the PROCESS macro (V3.4.2) for SPSS (model 4) with a level of confidence at 95% and 5000 bootstrap samples. Age, sex, years of education, disease duration HAMA and HAMD scores were controlled as covariates.

## 3 Results

### 3.1 Demographic and Clinical Characteristics

The demographic and clinical characteristics of the three groups are summarized in Table 1. No significant differences were observed among the groups in terms of age, sex, years of education, or disease duration. The OD group demonstrated significantly higher UPDRS‐III scores than the NOD group. Conversely, the OD group showed significantly lower scores on the MoCA and SST scores than both the NOD and HC groups. Differences in HAMA and HAMD scores between the NOD and OD groups were not statistically significant. Moreover, a positive correlation (*r* = 0.281, *p* = 0.003, Figure 1B) was observed between the MoCA and SST scores among PD patients, while a negative correlation (*r* = −0.285, *p* = 0.003, Figure S3A) was found between the UPDRS‐III and SST scores, after controlling for age, sex, years of education, and disease duration.

**TABLE 1:** Demographic and clinical characteristics of all subjects.

	PD‐NOD	PD‐OD	HC	*p*
	*n* = 36	*n* = 77	*n* = 51	
age (years)	57.81 ± 8.33	60.32 ± 7.69	59.63 ± 7.57	0.280^a^
sex (male) (%)	19 (52.78%)	45 (58.44%)	26 (50.98%)	0.680^b^
education (years)	10.69 ± 3.16	10.23 ± 2.84	10.45 ± 2.53	0.761^c^
Disease duration (years)	1.53 ± 1.28	2.45 ± 3.68	—	0.100^d^
UPDRS part III	15.61 ± 6.29	21.97 ± 8.76	—	**< 0.001** ^**d**^
MoCA	24.22 ± 2.73	21.73 ± 4.49	26.75 ± 1.70	**< 0.001** ^c^, ^f^
PDNMS	5.83 ± 3.27	7.97 ± 5.47	—	0.057^d^
HAMA	5.89 ± 4.97	6.16 ± 4.74		0.625^d^
HAMD	7.64 ± 5.63	8.84 ± 6.22		0.343^d^
SST	8.75 ± 0.84	4.55 ± 1.37	9.01 ± 0.96^h^	**< 0.001** ^c^, ^g^
**Cognitive domains (*z*‐score)**				
Attention/working memory	0.17 ± 0.62	−0.06 ± 0.88		0.210^e^
Executive	0.11 ± 0.75	−0.07 ± 0.58		0.231^e^
Visuospatial function	−0.07 ± 0.83	0.05 ± 0.88		0.539^e^
Memory	−0.02 ± 0.87	0.01 ± 0.68		0.883^e^
Language	−0.03 ± 0.87	0.02 ± 0.89		0.798^e^

*Note:* Data were expressed as mean ± SD or n (%). Statistically significant differences are shown in bold.

^a^ One‐way ANOVA.

^b^ Chi‐square test.

^c^ Kruskal–Wallis W test.

^d^ Mann−Whitney U test.

^e^ Two‐sample *t*‐tests.

^f^ The differences between OD and NOD, NOD and HC, as well as OD and HC are all significant.

^g^ The differences between OD and NOD, as well as between OD and HC, are both significant.

^h^ Eleven participants did not complete the test.

Abbreviations: HAMA, Hamilton Anxiety Scale; HAMD, Hamilton Depression Rating Scale; HC, healthy control; MoCA, The Montreal Cognitive Assessment; NOD, none‐olfactory dysfunction; OD, olfactory dysfunction; PD, Parkinson's disease; PDNMS, Parkinson disease Non‐Motor Symptoms Questionnaire; SST, Sniffin’ Sticks test; UPDRS‐III, Unified Parkinson's Disease Rating Scale, Part III.

### 3.2 Analysis Part I

#### 3.2.1 Network Graph Metrics

The averaged FC matrix for all participants and each group is illustrated in Figure S1. Most connections within the olfactory network were observed to be positive. The total number of significant connections was 528 for the HC group, 510 for the NOD group, and 533 for the OD group, all representing more than 90% of all possible network connections (561).

Over the sparsity ranging from 0.10 to 0.38, with a step of 0.01, the HC, OD, and NOD groups all exhibited a highly efficient small‐world topology (γ = Cp/Cr > 1, λ = Lp/Lr ≈ 1, and σ = γ/λ > 1; Figure S2). No significant differences were observed in γ, λ, σ, Lp, Cp, Eglob, Eloc, and other nodal metrics. As illustrated in Figure 1C, the left insula demonstrated a higher aBc in the NOD group (9.66 ± 8.11) compared to the OD group (4.93 ± 3.67) and the HC group (5.13 ± 4.37).

#### 3.2.2 Functional Connectivity Characteristics

**FIGURE 2:**
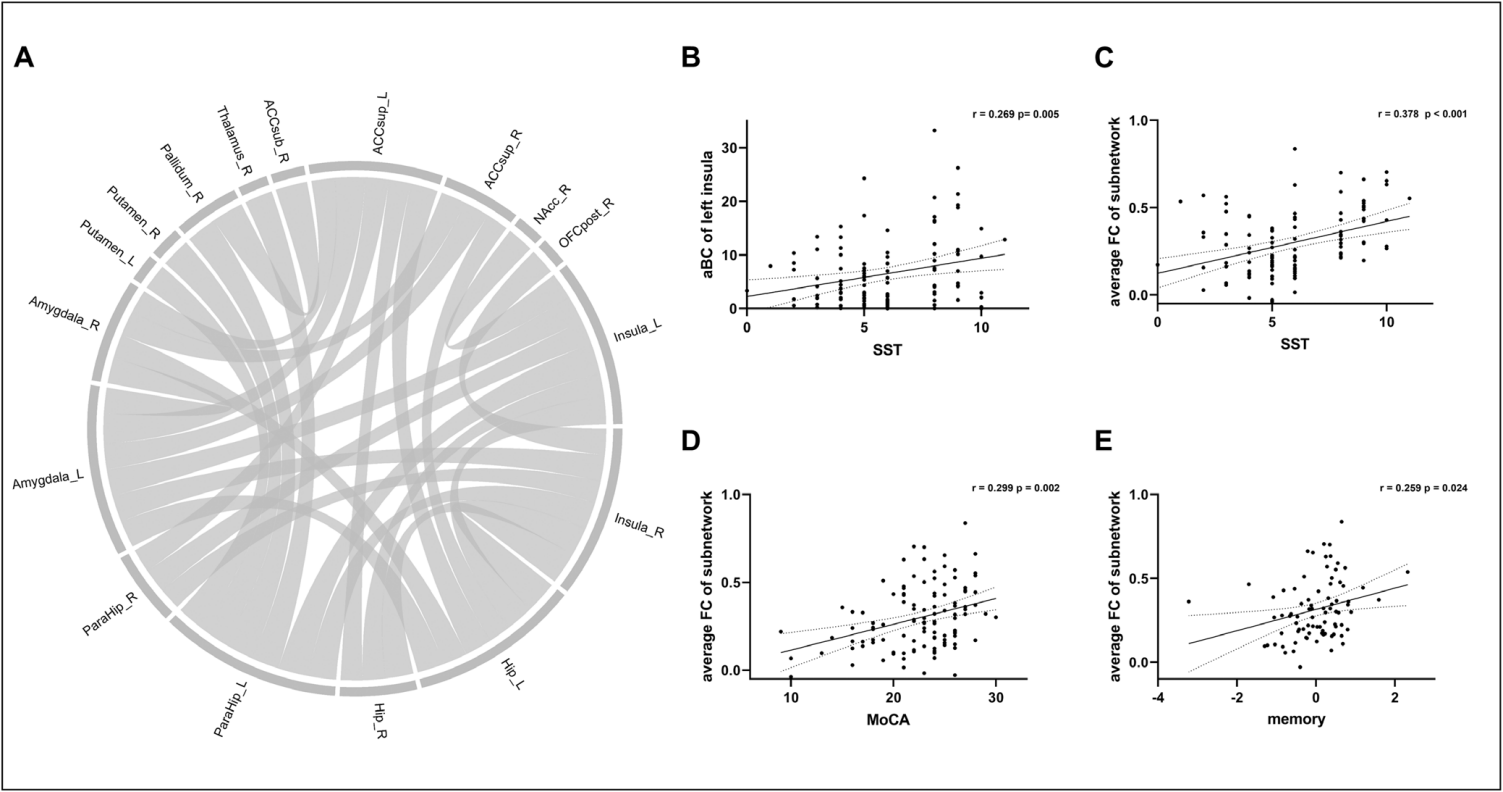
(A) The subnetwork exhibiting decreased functional connectivity in the OD group compared to the NOD group in Analysis Part I. (B) The correlation between the aBC of the left insula and the SST score. (C–E) The correlation between the average FC of the identified subnetwork and the SST, MoCA scores, and z‐score of the memory domain.

The NBS analysis identified a subnetwork (*p* = 0.011, NBS corrected, Figure 2A) that exhibited reduced FC within the olfactory network in the OD group compared to the NOD group. The subnetwork comprised 17 nodes, including the bilateral amygdala, parahippocampus, hippocampus, insula, ACC, putamen, right posterior olfactory cortex, nucleus accumbens, thalamus, and pallidum, as well as 29 edges. No alterations in FC within the olfactory network were observed in either the NOD or OD groups compared to the HC group.

#### 3.2.3 Correlation and Mediation Analyses (Patients Only)

**FIGURE 3:**
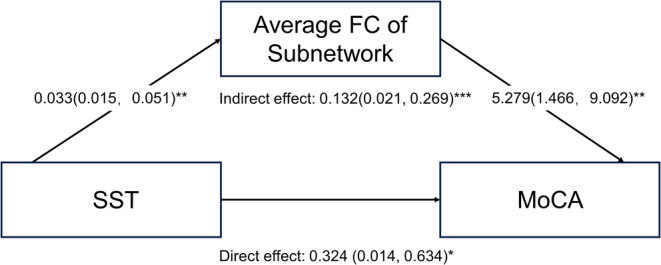
Mediation models with the average FC of the identified subnetwork as a mediator between SST and MoCA scores. *p < 0.05, **p < 0.01, **p < 0.001.

The aBc of the left insula was significantlycorrelated with the SST (*r* = 0.269, *p* = 0.005, Figure 2B) and UPDRS‐III scores (*r* = −0.258, *p* = 0.007, Figure S3B). Both SST (*r* = 0.378, *p* < 0.001, Figure 2C) and MoCA (*r* = 0.299, *p* = 0.002, Figure 2D) scores were positively correlated with the average FC of the identified subnetwork. Next, we conducted a mediation analysis to investigate whether the average FC of the altered olfactory subnetwork significantly mediated the relationship between olfactory and cognitive functions. The mediation model is shown in Figure 3. The total effect of the SST score on the MoCA score was significant (B = 0.456, CI = [0.164, 0.749]), as was the direct effect (B = 0.324, CI = [0.014, 0.634]) and the indirect effect (B = 0.132, CI = [0.021, 0.269]). These findings indicate that the effect of olfactory function on global cognitive function, as measured by MoCA, was partially mediated by the average FC of the altered olfactory network caused by OD. Among the five cognitive domains, memory was observed to be positively correlated with the average FC of the altered olfactory subnetwork (*r* = 0.259, *p* = 0.024, Figure 2E).

In addition, as illustrated in Figure S3C, the aBC of the left insula partially mediated the effect of olfactory function on motor symptom severity, as measured by UPDRS‐III. The total effect was significant (B = −1.079, CI = [−1.771, −0.386]), as were the direct (B = −0.883, CI = [−1.591, −0.175]) and indirect effects (B = −0.195, CI = [−0.466, −0.002]).

### 3.3 Analysis Part II

#### 3.3.1 Network Graph Metrics

The averaged FC matrix for all participants and each group is illustrated in Figure S4. Most connections within the olfactory network were observed to be positive. The total number of significant connections was 2499 for the HC group, 2245 for the NOD group, and 2610 for the OD group, all representing more than 75% of all possible network connections (3003).

Over the sparsity ranging from 0.06 to 0.30, with a step of 0.01, the HC, OD, and NOD groups all exhibited a highly efficient small‐world topology (γ = Cp/Cr > 1, λ = Lp/Lr ≈ 1, and σ = γ/λ > 1; Figure S5). No significant differences were observed in γ, σ, Lp, Cp, Eglob, Eloc, and other nodal metrics. The OD group demonstrated a higher normalized characteristic path length (λ) compared to the NOD groups (Figure S6B). In addition, the right ventral dysgranular and granular insula in the OD group demonstrated a lower nodal efficiency (Ne) compared to the HC and NOD groups (Figure S6C).

#### 3.3.2 Functional Connectivity Characteristics

The NBS analysis identified a subnetwork (*p* = 0.044, NBS corrected, Figure S7A) exhibiting reduced connectivity, which included 43 nodes and 70 edges. The nodes comprised subregions of the insula, cingulate gyrus, parahippocampus, nucleus accumbens, amygdala, hippocampus, and orbital gyrus (Table S2). No alterations in FC within the olfactory network were observed in either the NOD or OD groups compared to the HC group.

#### 3.3.3 Correlation and Mediation Analyses (Patients Only)

The λ was not correlated with the MoCA (*r* = −0.103, *p* = 0.289) or SST (*r* = −0.185, *p* = 0.056) scores. The aNe of the right ventral dysgranular and granular insula was positively correlated with only the SST score (*r* = 0.203, *p* = 0.036, Figure S7B). Although both the SST (*r* = 0.432, *p* < 0.001, Figure S7C) and MoCA (*r* = 0.237, *p* = 0.014, Figure S7D) scores were positively correlated with the average FC of the identified subnetwork, the indirect effect of the SST score on the MoCA score was no longer significant (B = 0.094, CI = [−0.038, 0.216]), indicating that the effect of olfactory function on global cognitive function was not mediated by the average FC of the altered olfactory network. Finally, none of the five cognitive domains was observed to be correlated with the average FC of the altered olfactory subnetwork.

## 4 Discussion

This study explored changes in the olfactory network associated with OD and its relationship to cognitive function in PD. Our findings indicate that, in PD patients with OD, the insula's role in integrating and communicating within the olfactory network was impaired, compared to those without OD. Network‐based statistical analysis revealed a disrupted subnetwork in PD patients with OD, characterized by reduced FC involving limbic brain regions, including the bilateral insula, amygdala, hippocampus, and anterior cingulate cortex. Notably, mediation analysis showed that the average FC of this subnetwork partially mediates the link between olfactory and cognitive functions.

OD is one of the earliest non‐motor symptoms in PD [32]. According to Braak's pathological staging model, pathological changes in the brains of PD patients initially occur in olfactory‐related regions, such as the olfactory bulb, anterior olfactory nucleus, and certain secondary olfactory structures [33, 34], explaining the common occurrence of OD in the early stage of PD. Therefore, OD may serve as an indicator of the severity of pathological changes in early stage of PD. In accordance with this, we found that PD patients with OD in our early‐stage cohort exhibited more severe motor symptoms and cognitive impairment compared to those without. Furthermore, the SST score exhibited a positive correlation with the MoCA score, consistent with previous research suggesting that more severe OD was associated with greater cognitive deficits and overall disease severity in PD patients [35], indicating that OD and cognitive impairment may involve damage to overlapping brain networks.

The insula is a region of the brain that has been increasingly recognized for its multifaceted roles in various high‐level brain functions, including emotional regulation, sensory integration, cognitive control, and olfactory processing [36]. Recent research has already confirmed the insula as one of the hub nodes in the human olfactory network [18]. Betweenness centrality is a metric used to evaluate the significance of a node within a network, with higher values indicating that the node acts as a bridge, facilitating communication between distinct regions of the brain. In our research, compared to the HC group, the NOD group exhibited higher betweenness centrality in the left insula, suggesting that the left insula may play an enhanced role in the integration of olfactory network information in early‐stage PD patients without OD, which may represent a compensatory mechanism. A previous study also reported that the betweenness centrality of the dorsal anterior insula (dAI) was increased in PD patients [37]. However, in PD patients with OD, the betweenness centrality of the left insula decreased, and this change partially mediated the relationship between olfactory performance and the severity of motor symptoms, suggesting that comorbid OD disrupted the overall structure of the olfactory network. This disruption, particularly the reduced compensatory role of the insula in information integration, is associated with more severe motor symptoms in these patients. Subsequent NBS analysis further revealed that OD decreased the FC of insula within the olfactory network, consistent with previous studies that have shown that PD patients with severe hyposmia exhibited reduced insular functional activity [16] and decreased insular FC [38, 39]. In summary, as a central hub of large‐scale neural systems [40], the insula plays a key role in information integration within the olfactory network, and changes in the number or strength of its connections are closely related to clinical symptoms of PD.

In our study, we also found that the average FC of the identified abnormal subnetwork is not only related to olfaction but also positively correlated with global cognitive function, especially memory. PD patients with dementia exhibited cortical thinning in the right insula and reduced FC from the right insula to the anterior cingulate gyrus [41]. The strength of this connection was reported to be positively correlated with cognitive performance PD [37]. Previous research has revealed that the insula is functionally connected to a wide range of limbic and neocortical brain regions [42], and the reduced FC between the amygdala and the insula was also correlated with cognitive impairments in PD [43]. The amygdala is another hub nodes of the human olfactory network and also contributes greatly to normal cognitive function. Previous research demonstrated that the PD patients with severe hyposmia showed decreased amygdala FC which was significantly correlated with both reduction of global cognitive function and severity of hyposmia [17]. Additionally, the hippocampus and parahippocampal gyrus are crucial for memory formation and retrieval, being key components of cognitive function [44], while the ACC is traditionally considered to play a significant role in attention [45, 46] and working memory [47], and olfactory function, particularly the ability to discriminate odors, is attention‐dependent [48]. Previous studies have repeatedly reported that reduced FC value and impaired structural connectivity of ACC is associated with cognitive deficits in various diseases, including PD [37, 49].

It is noteworthy that most of these brain regions within this subnetwork were components of the limbic system. This was consistent with previous studies showing that the human olfactory network consists of three subnetworks (the olfactory sensory, olfactory limbic and olfactory frontal subnetworks) [18]. Emotion regulation and memory are associated with the limbic system [50, 51]. Therefore, the OD in PD patients results in the abnormal functioning of the olfactory‐limbic subnetwork, which in turn affects their cognitive functions, especially the memory. Previous research has also demonstrated correlations between olfactory performance and Lewy body pathology within the limbic regions [52]. Moreover, OD was reported to be associated with the atrophy of limbic structures and cognitive decline in PD patients with OD [12]. The mediation analysis of our study indicated that the average FC of the decreased olfactory‐limbic subnetwork caused by OD partially mediates the relationship between olfactory and cognitive functions, providing further validation of the aforementioned viewpoint, providing valuable insights into the pathophysiology of PD and highlighting the importance of early OD as a potential biomarker for cognitive decline.

In the higher‐granularity olfactory network comprising 78 brain regions, the importance of the insula remained prominent, with the right insular subregion exhibited decreased nodal efficiency, which correlated significantly with olfactory function. Moreover, all insular subregions participated extensively in the identified abnormal subnetwork by contributing the greatest number of connections. This finding was consistent with the results of lower‐granularity network analysis, which underscored the insula's fundamental role in olfactory processing and integration. The observed increase in λ in the higher‐granularity olfactory network suggested a disruption in global network integration that was not apparent in the lower‐granularity analysis. This discrepancy may be attributed to the tendency of the lower‐granularity network to mask decreases in network efficiency by integrating signals from larger brain regions, enhancing the detection of long‐range connections but overlooking local changes [53]. In contrast, higher‐granularity networks capture more localized neural activity, revealing more realistic connectivity patterns, including local alterations that could affect network efficiency. Additionally, in higher‐granularity analysis, the average FC of the subnetwork no longer mediated the correlation between olfaction and cognition. This may be related to the increased number of nodes in the higher‐granularity network. Previous research indicated that finer granularity is advantageous for capturing localized neural activities. However, it may also lead to increased variability and a reduction in signal strength, which may affect the detection of mediation effects [53]. Therefore, when conducting network analysis, it is crucial to balance the advantages and limitations of different granularities to obtain the most comprehensive understanding of the olfactory network.

AD and PD, the two most common neurodegenerative diseases, exhibit similar OD and olfactory‐related brain regions pathology in the early stage [54], although there are largely phenotypically and pathologically distinctive. Additionally, inhaled factors such as viruses, aerosolized metals, and toxins are also pathogenic risk factors for PD and AD [55]. Therefore, “olfactory vector hypothesis” was put forward that disease risk factors can enter the brain via the olfactory mucosa, and OD is regarded as the initiation of various neurodegenerative diseases [9]. So, it is imperative to focus research efforts on olfactory function. Furthermore, as olfactory impairment serves as a harbinger for cognitive decline, our research revealed the possible neural mechanisms connecting olfaction and cognition. This provides a theoretical basis for olfactory training as a prospective means to enhance cognitive functions [2], proposing a novel preventive strategy against cognitive impairment.

## 5 Limitations

Several limitations should be considered when interpreting our results. First, this was a cross‐sectional study, limiting causal inferences about the temporal ordering between olfactory loss, connectivity changes and cognitive decline. Longitudinal research is needed. Second, the sample size was relatively small, focusing only on PD patients in the early stage. Larger multicenter studies across wider disease spectrum are warranted to better validate the mechanism linking olfactory and cognitive performance. Third, we did not include dopaminergic imaging with SPECT or PET as PD diagnosis criteria. Fourth, although we have attempted to study the olfactory network at a higher granularity, the relatively small sample size restricted the statistical power and hindered our ability to investigate the more subtle changes in the olfactory network and its correlation with the clinical symptoms of PD.

## 6 Conclusion

In conclusion, this study underscores the pivotal role of the insula in the olfactory network of PD patients, particularly in those with OD. The disruption in the olfactory‐limbic subnetwork, with the insula at its core, not only correlates with olfactory impairment but also mediates its impact on cognitive functions. These findings highlight the potential of early OD as a biomarker for cognitive decline in PD, emphasizing the need for further research into olfactory training as a preventive strategy against cognitive impairment.

## Author Contributions

W.L. organized the project and critically revised the manuscript. Y.X. organized the project, drafted the preliminary manuscript, collected data, and performed statistical analysis. H.Z. collected data and performed statistical analysis. S.C., Y.C., and J.L. collected data. W.L. and J.R. critiqued the statistical analysis and interpreted the data. All authors contributed to the article and approved the submitted manuscript.

## Ethics Statement

The Medical Ethics Committee of the Affiliated Brain Hospital of Nanjing Medical University provided ethical approval for this study.

## Consent

Written informed consent was obtained from all the patients before study participation.

## Conflicts of Interest

The authors declare no conflicts of interest.

## Supporting information


Supplement material

## Data Availability

The original data of this study can be obtained from the corresponding author upon reasonable request.
