# Supplementary material for: Olfactory Network Disruptions as Mediators of Cognitive Impairment in De Novo Parkinson's Disease
Source: CNS Neurosci Ther. 2025 Jan 13;31(1):e70198. doi: 10.1111/cns.70198 (PMC11726119; doi:10.1111/cns.70198)
Supplement: Supplementary file 1 — Supplement material [file CNS-31-e70198-s001.docx]

**
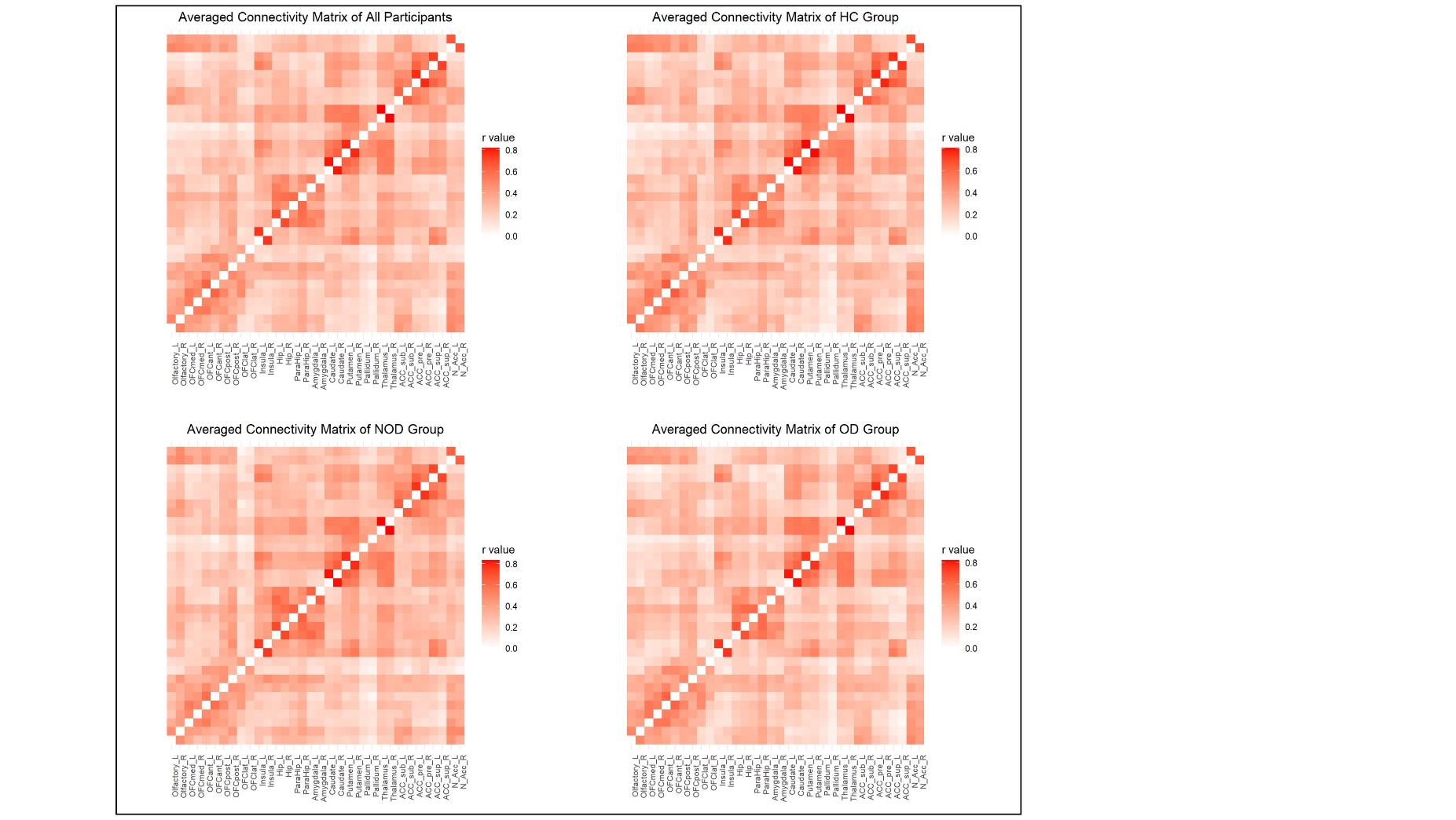
FIGURE S1** The averaged FC matrix for all participants and each group in Analysis Part I.


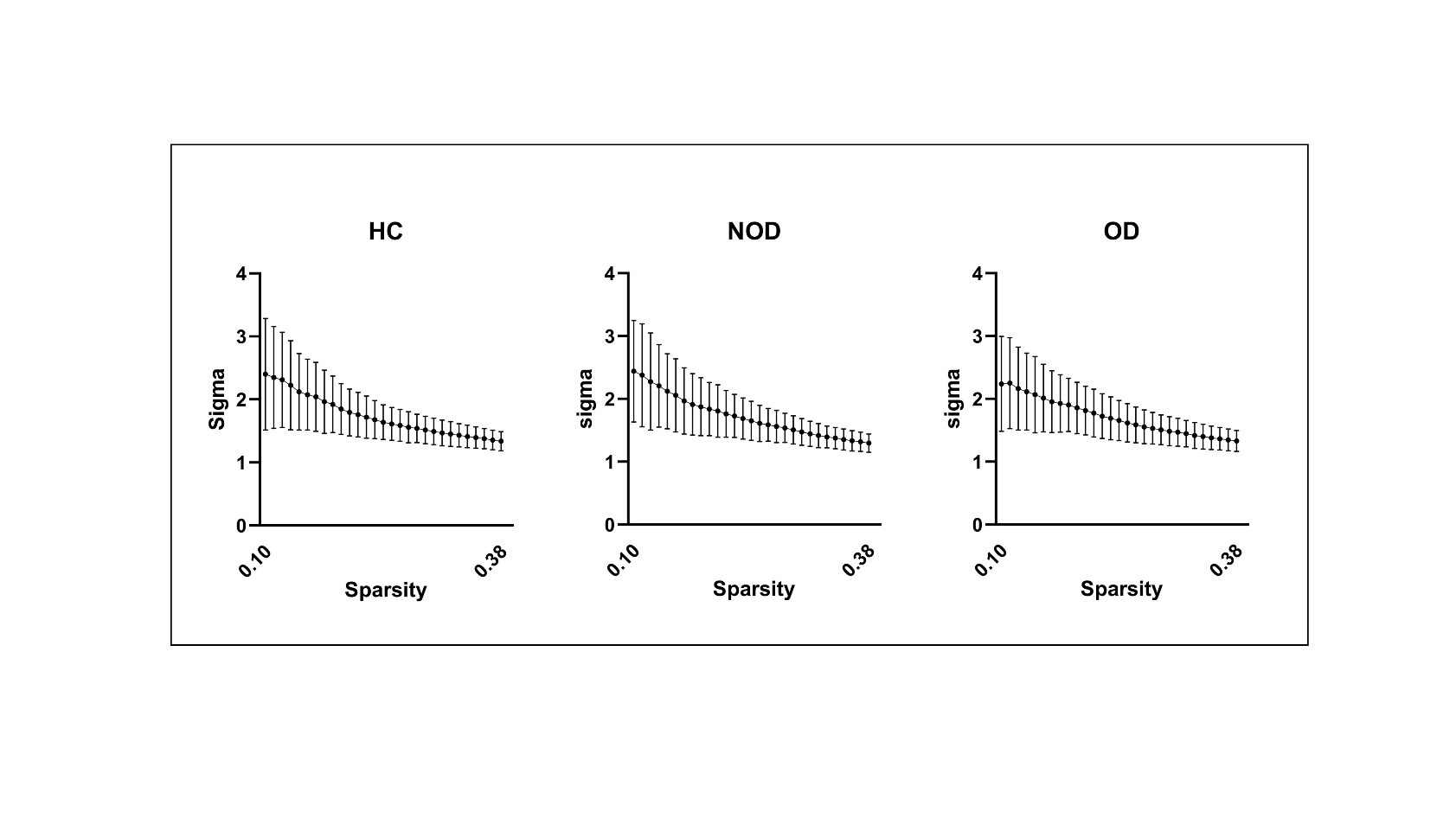
**FIGURE S2** The sigma for each group under a sparsity range of 0.10 to 0.38 in Analysis Part I.


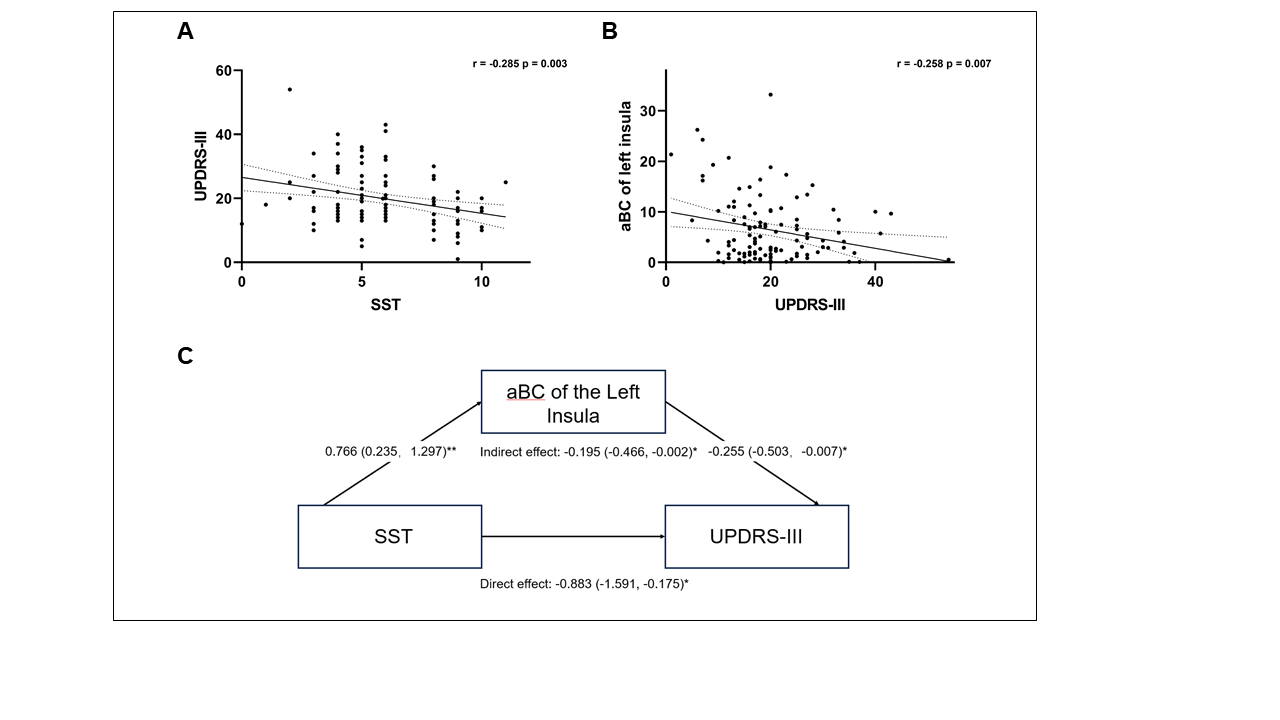


**FIGURE S3 A** The correlation between the SST and UPDRS-III scores. **B** The correlation between the aBC of left insula and the UPDRS-III score. **C** Mediation models with the aBC of left insula as a mediator between the SST and UPDRS-III scores. * p < 0.05, ** p < 0.01, ** p＜0.001.


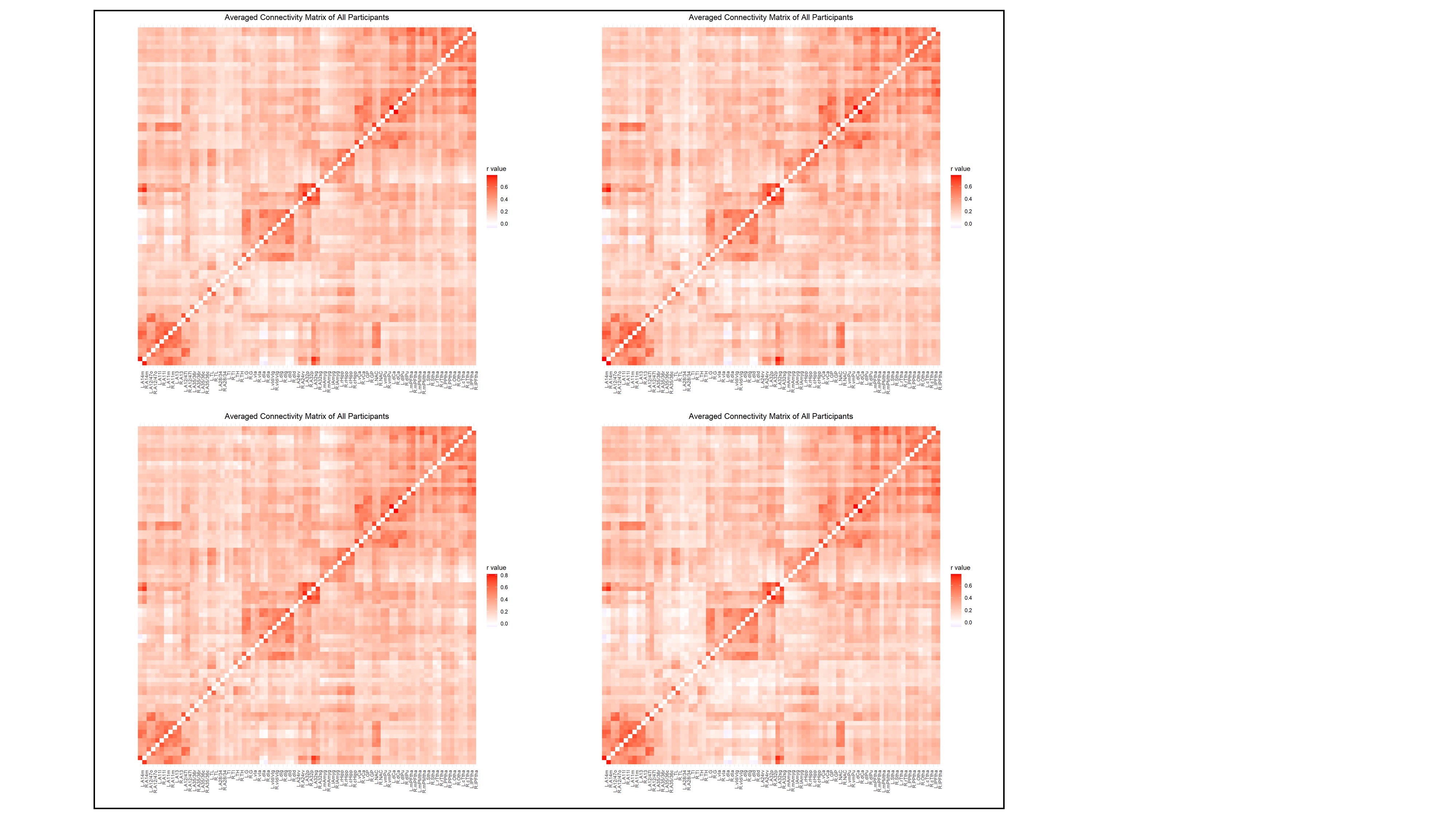
**FIGURE S4** The averaged FC matrix for all participants and each group in Analysis Part Ⅱ.


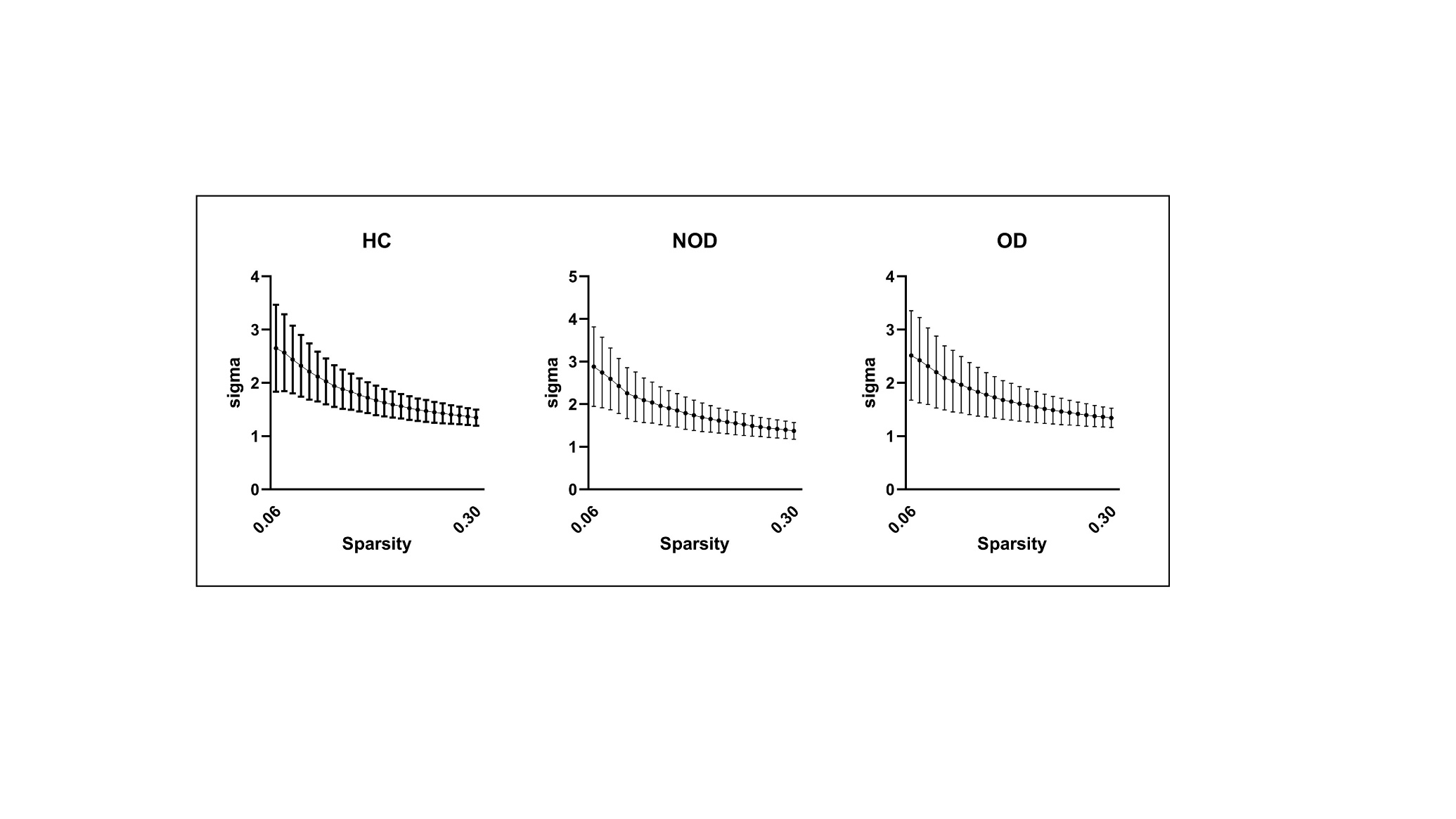
**FIGURE S5** The sigma for each group under a sparsity range of 0.06 to 0.30 in Analysis Part Ⅱ.


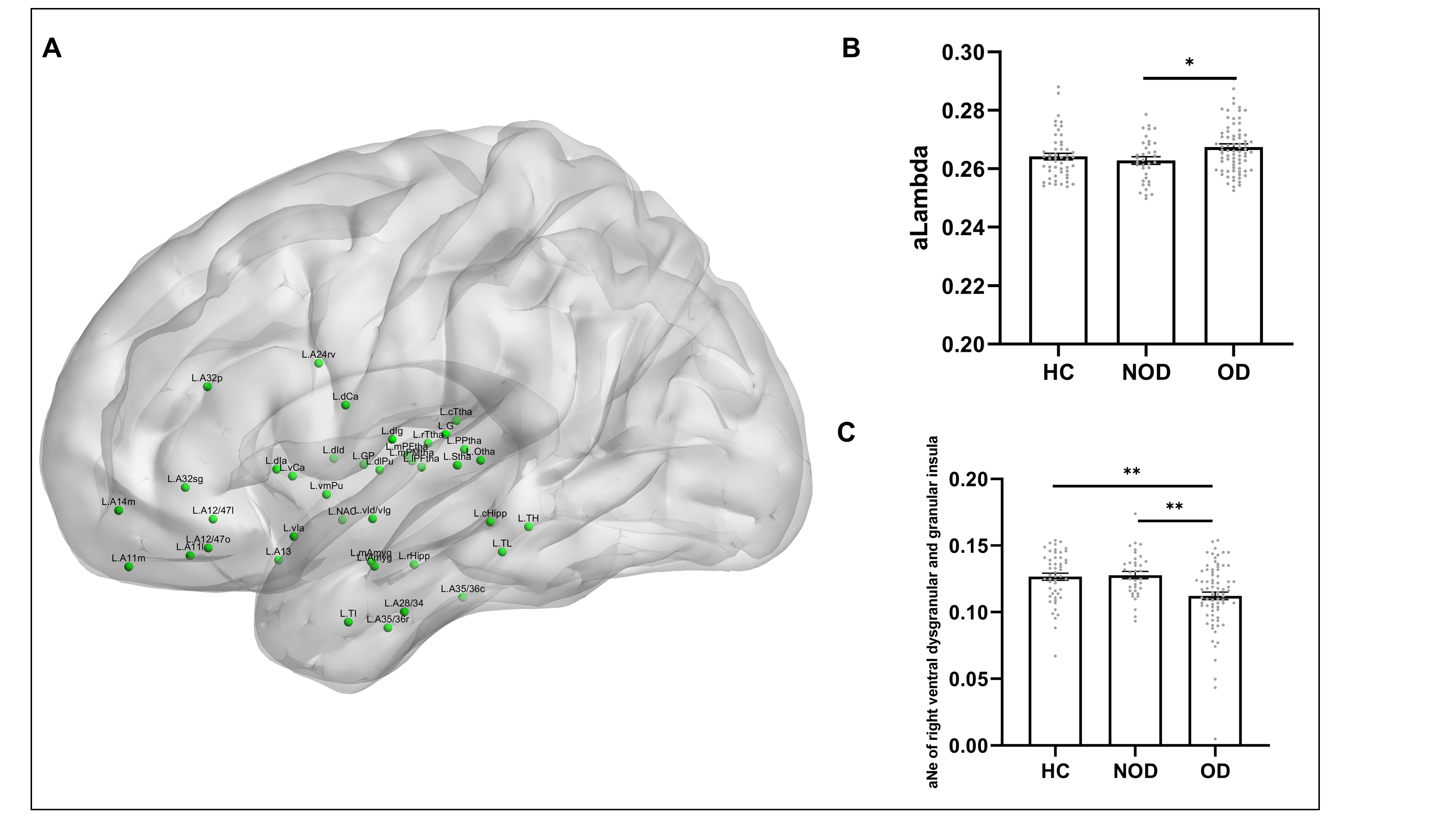
**FIGURE S6** **A** Unilateral 39 nodes in olfactory network. **B** The intergroup differences in the lambda. **C** The intergroup differences in the aNe of the right ventral dysgranular and granular insula. * p < 0.05, ** p < 0.01, ** p＜0.001.


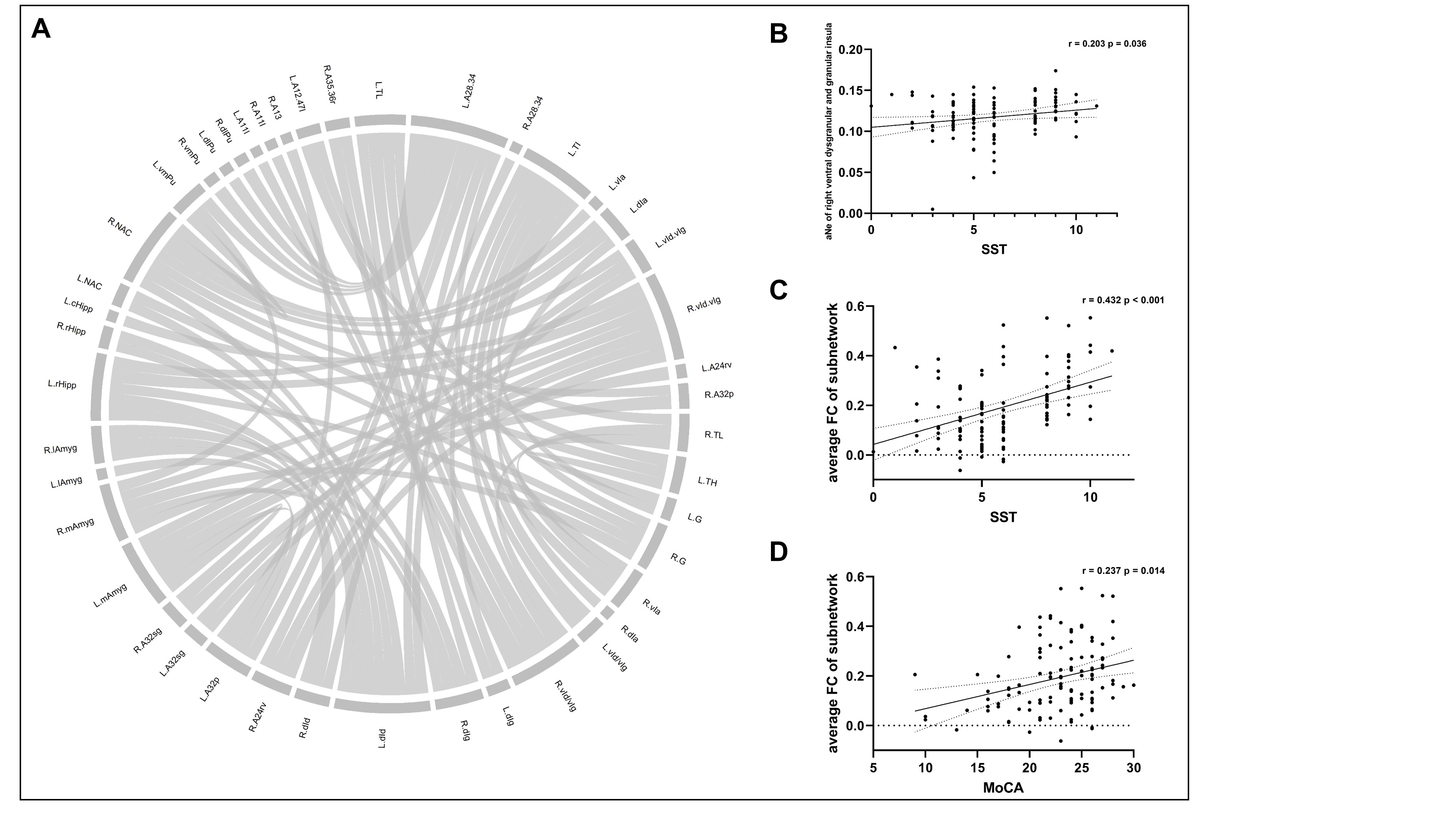
**FIGURE S7** **A** The subnetwork exhibiting decreased functional connectivity in the OD group compared to the NOD group in Analysis Part Ⅱ. **B** The correlation between the aNe of the right ventral dysgranular and granular insula and the SST. **CD** The correlation between the average FC of the identified subnetwork and the SST, and MoCA scores.

**Table S1** Olfactory-Related Brain Regions in the Brainnetome Atlas

| Label ID | Gyrus | Abbr. | Anatomical and modified Cyto-architectonic descriptions |
| --- | --- | --- | --- |
| 41 | Orbital Gyrus | L.A14m | L. medial area 14 |
| 42 | Orbital Gyrus | R.A14m | R. medial area 14 |
| 43 | Orbital Gyrus | L.A12/47o | L. orbital area 12/47 |
| 44 | Orbital Gyrus | R.A12/47o | R. orbital area 12/47 |
| 45 | Orbital Gyrus | L.A11l | L. lateral area 11 |
| 46 | Orbital Gyrus | R.A11l | R. lateral area 11 |
| 47 | Orbital Gyrus | L.A11m | L. medial area 11 |
| 48 | Orbital Gyrus | R.A11m | R. medial area 11 |
| 49 | Orbital Gyrus | L.A13 | L. area 13 |
| 50 | Orbital Gyrus | R.A13 | R. area 13 |
| 51 | Orbital Gyrus | L.A12/47l | L. lateral area 12/47 |
| 52 | Orbital Gyrus | R.A12/47l | R. lateral area 12/47 |
| 109 | Parahip | L.A35/36r | L. rostral area 35/36 |
| 110 | Parahip | R.A35/36r | R. rostral area 35/36 |
| 111 | Parahip | L.A35/36c | L. caudal area 35/36 |
| 112 | Parahip | R.A35/36c | R. caudal area 35/36 |
| 113 | Parahip | L.TL | L. area TL (lateral PPHC, posterior parahippocampal gyrus) |
| 114 | Parahip | R.TL | R. area TL (lateral PPHC, posterior parahippocampal gyrus) |
| 115 | Parahip | L.A28/34 | L. area 28/34 (EC,entorhinal cortex) |
| 116 | Parahip | R.A28/34 | R. area 28/34 (EC,entorhinal cortex) |
| 117 | Parahip | L.TI | L. area TI(temporal agranular insular cortex) |
| 118 | Parahip | R.TI | R. area TI(temporal agranular insular cortex) |
| 119 | Parahip | L.TH | L. area TH (medial PPHC) |
| 120 | Parahip | R.TH | R. area TH (medial PPHC) |
| 163 | Insular | L.G | L. hypergranular insula |
| 164 | Insular | R.G | R. hypergranular insula |
| 165 | Insular | L.vIa | L. ventral agranular insula |
| 166 | Insular | R.vIa | R. ventral agranular insula |
| 167 | Insular | L.dIa | L. dorsal agranular insula |
| 168 | Insular | R.dIa | R. dorsal agranular insula |
| 169 | Insular | L.vId/vIg | L. ventral dysgranular and granular insula |
| 170 | Insular | R.vId/vIg | R. ventral dysgranular and granular insula |
| 171 | Insular | L.dIg | L. dorsal granular insula |
| 172 | Insular | R.dIg | R. dorsal granular insula |
| 173 | Insular | L.dId | L. dorsal dysgranular insula |
| 174 | Insular | R.dId | R. dorsal dysgranular insula |
| 177 | Cingulate Gyrus | L.A24rv | L. rostroventral area 24 |
| 178 | Cingulate Gyrus | R.A24rv | R. rostroventral area 24 |
| 179 | Cingulate Gyrus | L.A32p | L. pregenual area 32 |
| 180 | Cingulate Gyrus | R.A32p | R. pregenual area 32 |
| 187 | Cingulate Gyrus | L.A32sg | L. subgenual area 32 |
| 188 | Cingulate Gyrus | R.A32sg | R. subgenual area 32 |
| 211 | Amygdala | L.mAmyg | L. medial amygdala |
| 212 | Amygdala | R.mAmyg | R. medial amygdala |
| 213 | Amygdala | L.lAmyg | L. lateral amygdala |
| 214 | Amygdala | R.lAmyg | R. lateral amygdala |
| 215 | Hippocampus | L.rHipp | L. rostral hippocampus |
| 216 | Hippocampus | R.rHipp | R. rostral hippocampus |
| 217 | Hippocampus | L.cHipp | L. caudal hippocampus |
| 218 | Hippocampus | R.cHipp | R. caudal hippocampus |
| 219 | Caudate | L.vCa | L. ventral caudate |
| 220 | Caudate | R.vCa | R. ventral caudate |
| 221 | Globus Pallidus | L.GP | L. globus pallidus |
| 222 | Globus Pallidus | R.GP | R. globus pallidus |
| 223 | Nucleus Accumbens | L.NAC | L. nucleus accumbens |
| 224 | Nucleus Accumbens | R.NAC | R. nucleus accumbens |
| 225 | Putamen | L.vmPu | L. ventromedial putamen |
| 226 | Putamen | R.vmPu | R. ventromedial putamen |
| 227 | Caudate | L.dCa | L. dorsal caudate |
| 228 | Caudate | R.dCa | R. dorsal caudate |
| 229 | Putamen | L.dlPu | L. dorsolateral putamen |
| 230 | Putamen | R.dlPu | R. dorsolateral putamen |
| 231 | Thalamus | L.mPFtha | L. medial pre-frontal thalamus |
| 232 | Thalamus | R.mPFtha | R. medial pre-frontal thalamus |
| 233 | Thalamus | L.mPMtha | L. pre-motor thalamus |
| 234 | Thalamus | R.mPMtha | R. pre-motor thalamus |
| 235 | Thalamus | L.Stha | L. sensory thalamus |
| 236 | Thalamus | R.Stha | R. sensory thalamus |
| 237 | Thalamus | L.rTtha | L. rostral temporal thalamus |
| 238 | Thalamus | R.rTtha | R. rostral temporal thalamus |
| 239 | Thalamus | L.PPtha | L. posterior parietal thalamus |
| 240 | Thalamus | R.PPtha | R. posterior parietal thalamus |
| 241 | Thalamus | L.Otha | L. occipital thalamus |
| 242 | Thalamus | R.Otha | R. occipital thalamus |
| 243 | Thalamus | L.cTtha | L. caudal temporal thalamus |
| 244 | Thalamus | R.cTtha | R. caudal temporal thalamus |
| 245 | Thalamus | L.lPFtha | L. lateral pre-frontal thalamus |
| 246 | Thalamus | R.lPFtha | R. lateral pre-frontal thalamus |

**Table S2** Nodes in the Abnormal Subnetwork Identified by NBS Analysis

| Label ID | Gyrus | Abbr. | Anatomical and modified Cyto-architectonic descriptions |
| --- | --- | --- | --- |
| 42 | Orbital Gyrus | R.A14m | R. medial area 14 |
| 45 | Orbital Gyrus | L.A11l | L. lateral area 11 |
| 46 | Orbital Gyrus | R.A11l | R. lateral area 11 |
| 50 | Orbital Gyrus | R.A13 | R. area 13 |
| 51 | Orbital Gyrus | L.A12/47l | L. lateral area 12/47 |
| 110 | Parahip | R.A35/36r | R. rostral area 35/36 |
| 113 | Parahip | L.TL | L. area TL (lateral PPHC, posterior parahippocampal gyrus) |
| 114 | Parahip | R.TL | R. area TL (lateral PPHC, posterior parahippocampal gyrus) |
| 115 | Parahip | L.A28/34 | L. area 28/34 (EC,entorhinal cortex) |
| 116 | Parahip | R.A28/34 | R. area 28/34 (EC,entorhinal cortex) |
| 117 | Parahip | L.TI | L. area TI(temporal agranular insular cortex) |
| 119 | Parahip | L.TH | L. area TH (medial PPHC) |
| 163 | Insular | L.G | L. hypergranular insula |
| 164 | Insular | R.G | R. hypergranular insula |
| 165 | Insular | L.vIa | L. ventral agranular insula |
| 166 | Insular | R.vIa | R. ventral agranular insula |
| 167 | Insular | L.dIa | L. dorsal agranular insula |
| 168 | Insular | R.dIa | R. dorsal agranular insula |
| 169 | Insular | L.vId/vIg | L. ventral dysgranular and granular insula |
| 170 | Insular | R.vId/vIg | R. ventral dysgranular and granular insula |
| 171 | Insular | L.dIg | L. dorsal granular insula |
| 172 | Insular | R.dIg | R. dorsal granular insula |
| 173 | Insular | L.dId | L. dorsal dysgranular insula |
| 174 | Insular | R.dId | R. dorsal dysgranular insula |
| 177 | Cingulate Gyrus | L.A24rv | L. rostroventral area 24 |
| 178 | Cingulate Gyrus | R.A24rv | R. rostroventral area 24 |
| 179 | Cingulate Gyrus | L.A32p | L. pregenual area 32 |
| 180 | Cingulate Gyrus | R.A32p | R. pregenual area 32 |
| 187 | Cingulate Gyrus | L.A32sg | L. subgenual area 32 |
| 188 | Cingulate Gyrus | R.A32sg | R. subgenual area 32 |
| 211 | Amygdala | L.mAmyg | L. medial amygdala |
| 212 | Amygdala | R.mAmyg | R. medial amygdala |
| 213 | Amygdala | L.lAmyg | L. lateral amygdala |
| 214 | Amygdala | R.lAmyg | R. lateral amygdala |
| 215 | Hippocampus | L.rHipp | L. rostral hippocampus |
| 216 | Hippocampus | R.rHipp | R. rostral hippocampus |
| 217 | Hippocampus | L.cHipp | L. caudal hippocampus |
| 223 | Nucleus Accumbens | L.NAC | L. nucleus accumbens |
| 224 | Nucleus Accumbens | R.NAC | R. nucleus accumbens |
| 225 | Putamen | L.vmPu | L. ventromedial putamen |
| 226 | Putamen | R.vmPu | R. ventromedial putamen |
| 229 | Putamen | L.dlPu | L. dorsolateral putamen |
| 230 | Putamen | R.dlPu | R. dorsolateral putamen |
